# Supplementary material for: Gene Suppression Therapies in Hereditary Cerebellar Ataxias: A Systematic Review of Animal Studies
Source: Cells. 2023 Mar 29;12(7):1037. doi: 10.3390/cells12071037 (PMC10093402; doi:10.3390/cells12071037)
Supplement: Supplementary file 1 [file cells-12-01037-s001.zip › cells-2216638-supplementary.pdf]

Supplementary Materials

Table S1- Standardized Sheet for Data Extraction

Author	Year	Type HCA	Type GST	Aim	Intervention				Categories Analysed			Outcome Results		Key conclusions of study authors
					Control 1	Control 2	Control 3	Others	Suppression efficacy	Motor Behavior	Safety Profile	Outcome	Overall Results	
